# Efficacy of a spot-on formulation containing moxidectin 2.5%/imidacloprid 10% for the treatment of *Cercopithifilaria* spp. and *Onchocerca lupi* microfilariae in naturally infected dogs from Portugal

**DOI:** 10.1186/s13071-021-04704-7

**Published:** 2021-04-13

**Authors:** Domenico Otranto, Vito Colella, Marcos Antônio Bezerra-Santos, Jairo Alfonso Mendoza-Roldan, Maria Alfonsa Cavalera, André Pereira, Roland Schaper, Carla Maia

**Affiliations:** 1 Department of Veterinary Medicine, University of Bari, Valenzano, 70010 Italy; 2 Department of Pathobiology, Faculty of Veterinary Science, Bu-Ali Sina University, Hamedan, Iran; 3 Faculty of Veterinary and Agricultural Sciences, University of Melbourne, Parkville, Australia; 4 Global Health and Tropical Medicine (GHTM), Instituto de Higiene E Medicina Tropical (IHMT), Universidade NOVA de Lisboa (NOVA), Lisboa, Portugal; 5 Elanco Animal Health, Monheim, Germany

**Keywords:** Dogs, *Onchocerca lupi*, *Cercopithifilaria* spp., Treatment, Moxidectin, Imidacloprid

## Abstract

**Background:**

*Onchocerca lupi* and *Cercopithifilaria* spp. are vector-borne filarioids of dogs, which harbour skin microfilariae (mfs), the former being of zoonotic concern. Proper treatment studies using compounds with microfilaricidal activity have not been performed. Therefore, this study aimed to assess the efficacy of a commercially available spot-on formulation containing moxidectin 2.5%/imidacloprid 10% for the treatment of *O. lupi* or *Cercopithifilaria* spp. skin-dwelling mfs in naturally infected dogs.

**Methods:**

Privately owned dogs (*n* = 393) from southern Portugal were sampled via skin biopsies to identify and count mfs in 20 µl of skin sediment. A total of 22 mfs-positive dogs were allocated to treatment group (*n* = 11; G1) or left untreated as a control (*n* = 11; G2). As a pilot investigation to test the treatment efficacy, five dogs assigned to G1 were treated four times at monthly intervals with moxidectin 2.5%/imidacloprid 10% spot-on formulation on SDs 0, 28 (± 2), 56 (± 2), and 84 (± 2). Based on the negative results for both *O. lupi* and/or *Cercopithifilaria* spp. mfs of dogs in the pilot study from SD28 onwards, the remaining six dogs in G1 were treated at SD0 and assessed only at SD28.

**Results:**

Of the 393 animals sampled, 78 (19.8%) scored positive for skin-dwelling mfs. At the pilot investigation, a mean number of 19.6 mfs for *O. lupi* was recorded among five infected dogs whereas no mfs were detected at SD28. At SD0, the mean number of *Cercopithifilaria* spp. larvae was 12.6 for G1 and 8.7 for G2. The mean number of mfs for G2 was 20.09.

**Conclusions:**

Results herein obtained suggest that a single treatment with moxidectin 2.5%/imidacloprid 10% spot-on formulation is efficacious against skin-dwelling mfs in dogs. The microfilaricidal effect of moxidectin could also be useful in reducing the risk of *O. lupi* infection for humans.
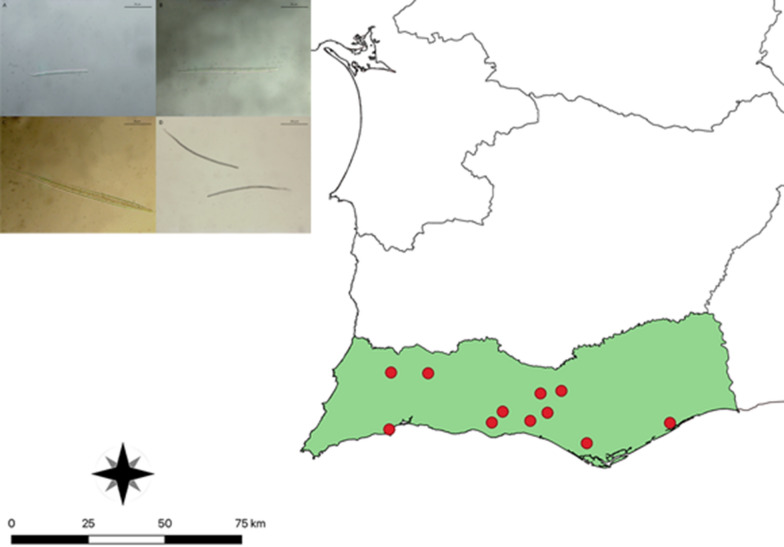

## Background

Vector-borne filarioids are transmitted by a wide range of arthropods (e.g., mosquitoes, fleas, blackflies, biting midges and ticks). About 12 species belonging to the genera *Acanthocheilonema*, *Brugia*, *Cercopithifilaria*, *Dipetalonema*, *Dirofilaria*, and *Onchocerca* infect dogs [1–4]. Among these, the species *Dirofilaria repens* and *Dirofilaria immitis* are the best-known filarioids in veterinary parasitology for their worldwide distribution and their zoonotic potential [5–7]. The genus *Onchocerca* includes 30 species primarily infecting ungulates [8] and, to a lesser extent, carnivores and humans. In the last decade, the increased number of zoonotic cases of *O. lupi* has stimulated the interest of the scientific community in this little-known parasite [9–11]. Since the first molecularly confirmed case of infection by *O. lupi* in humans [9], to date, 22 patients have been diagnosed with this onchocercid species in Germany, Tunisia, Hungary, Greece, Turkey, Iran, and the USA [12–15]. Consequently, much more attention is currently given to the distribution and clinical significance of *O. lupi* in dogs. In the initial stage, canine onchocercosis is associated with nonspecific ocular discomfort (i.e. excessive lacrimation, photophobia, conjunctivitis, exophthalmos, and periorbital swelling), while in the chronic phase the typical nodules are detected on the external parts of the ocular apparatus (i.e. eyelids, nictitating membrane, conjunctiva, and sclera) [16]. However, dogs do not always display overt clinical signs when *O. lupi* adults localize in the retrobulbar space of the eye [17]. Moreover, adult nematodes may localise in other anatomical districts other than the ocular apparatus, given that microfilariae (mfs) were diagnosed in dogs with absence of nodules together with the lack of ocular lesions at ultrasound examination [18].

The arthropod species involved in the life cycle of *O. lupi* have not been identified so far. Blackflies of the species *Simulium velutinum, Simulium reptans*, and *Simulium pseudequinum*, which fed on an *O. lupi*-infected dog from Greece, did not demonstrate progression of nematode larval development [19]. However, *O. lupi* DNA has been found in *Simulium tribulatum* blackflies from the USA [20].

Dogs may also be infected by at least three species of *Cercopithifilaria*, namely *Cercopithifilaria grassii*, *Cercopithifilaria* sp. II sensu Otranto et al. 2013 (a formal taxonomic description of adults for this species is lacking), and *C. bainae* [21]. In particular, *C. bainae* is prevalent in canine and tick populations from Southern and Eastern Europe (i.e. Spain, Italy, Greece, Portugal, and Romania) [22–24]. In addition, the parasite has been reported in Australia, Brazil, Malaysia, South Africa, and the USA [25, 26]. Generally, the parasite distribution overlaps that of its vector, the brown dog tick *Rhipicephalus sanguineus* sensu lato [27–29]. However, *C. bainae* is considered of minor importance in veterinary medicine with few reports of erythematous, papular, and pruritic dermatitis [21, 26] and chronic polyarthritis [30] associated with the infection in dogs.

The diagnosis of canine onchocercosis and cercopithifilarioses is achieved by the microscopic detection and identification of mfs in skin biopsies [9, 31, 32] or PCR-based techniques [9, 33, 34]. Preliminary investigations to develop tools able to detect anti-*O. lupi* antibodies have also been carried out [35–37]. Treatment of clinical cases of canine onchocercosis has been attempted using combinations of melarsomine, ivermectin, topical and systemic antibiotics, and prednisone [15, 16, 38]. A single dog presenting erythematous lesions and affected by *C. bainae* was successfully treated with a spot-on formulation containing imidacloprid 10% and moxidectin 2.5% [26]. In addition, the administration of oxfendazole (50 mg/kg/PO for 5 or 10 consecutive days) in dogs was ineffective in reducing skin-dwelling *O. lupi* mfs and showed a not statistically significant reduction of 50% of ocular lesions in a 6-month follow-up period [39]. However, proper studies on the long-term outcomes of macro- and microfilaricidal therapies for treating both parasites have not yet been performed, and no indications for the best treatment protocol to use are available. We assessed the efficacy of a commercially available spot-on formulation containing moxidectin and imidacloprid for the treatment of *O. lupi* or *Cercopithifilaria* spp. skin-dwelling mfs in naturally infected dogs.

## Materials and methods

### Ethics statement

The trial was a negative control, and clinical efficacy study partially blinded using a randomized block design, conducted according to the standards of Good Scientific Practice (GSP) and the national animal welfare requirements. Privately owned dogs, which lived in an area endemic for *O. lupi* and *Cercopithifilaria* spp. of the Algarve region (southern Portugal), were enrolled [39, 40]. At the end of the study all dogs were treated with Advocate® spot-on (Bayer Animal Health) and all screened dogs were dewormed with Drontal® Plus (Bayer Animal Health).

### Sampling procedure, enrolment and follow-up

**Fig. 1:**
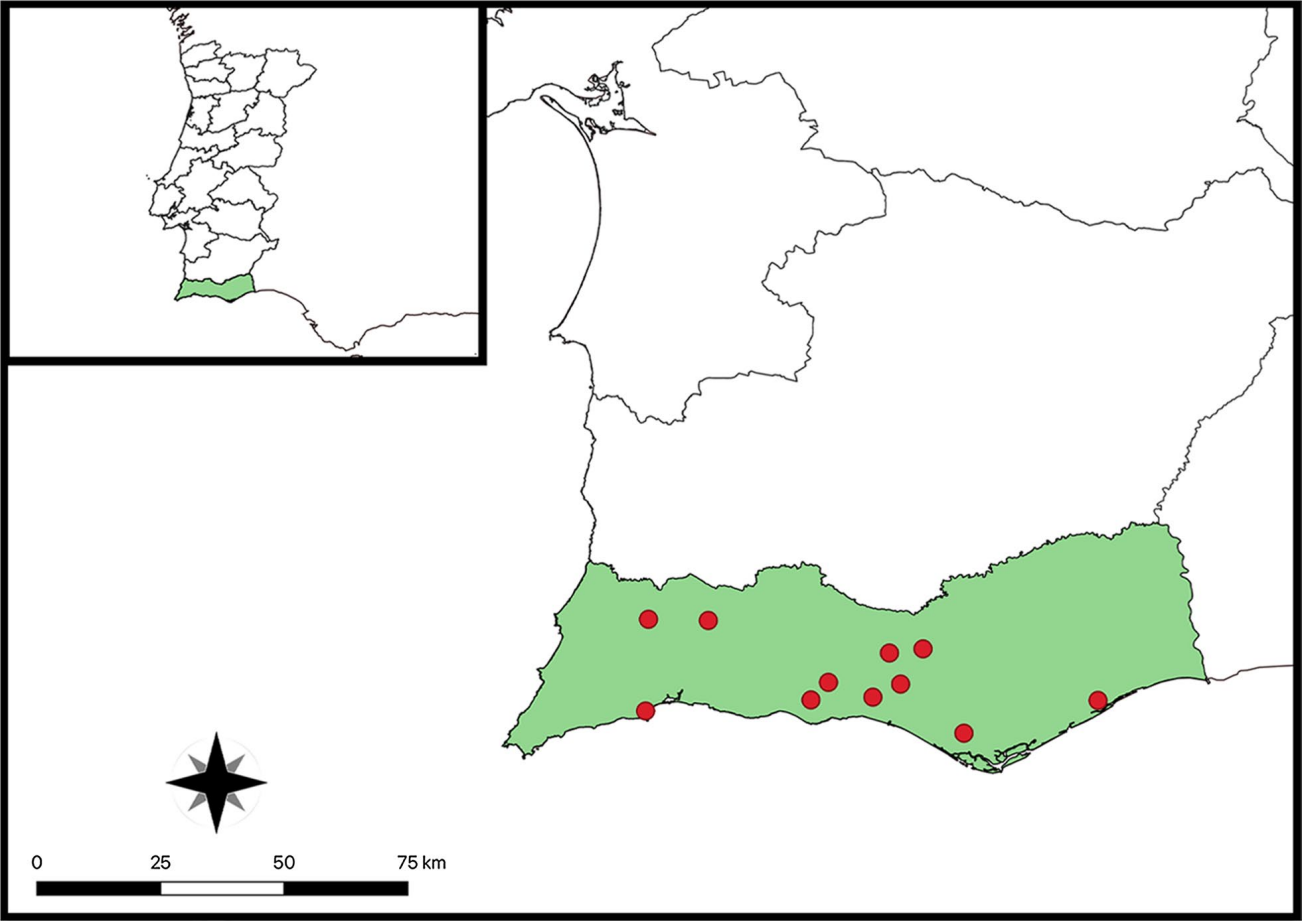
Map of the three areas in Algarve, Portugal, where the study was conducted (insert up left part) with a detail of the localities from where dogs were sampled (red dots)

From May to December 2018, 393 privately owned dogs (i.e. 242 males and 151 females) were sampled via skin biopsies for diagnosing *O. lupi* and *Cercopithifilaria* spp. infection. All animals came from the municipalities of southern Portugal, i.e. Benafim (latitude: 37°13′N; longitude: 8°07′W), Boliqueime (latitude: 37°08′N; longitude: 8°09′W), Guia (latitude: 37°07′N; longitude: 8°17′W), Lagos (latitude: 37°09′N; longitude: 8°44′W), Marmelete (latitude: 37°18′N; longitude: 8°40′W), Mata Lobos (latitude: 37°08′N; longitude: 8°01′W), Monte Seco (latitude: 37°10′N; longitude: 8°05′W), Parragil (latitude: 37°9′N; longitude: 8°05′W), Patacão (latitude: 37°3′N; longitude: 7°57′W), Picota (latitude: 37°09′N; longitude: 8°06′W), Salir (latitude: 37°14′N; longitude: 8°03′W), Tavira (latitudine: 37°07′N; longitudine: 7°38′W), and Tunes (latitude: 37°10′N; longitude: 8°15′W) (Fig. 1). Inclusion criteria were living outdoors or having regular outdoor activities and being at least 8 weeks of age and weighing > 1 kg. Dogs were excluded when they were treated with macrocyclic lactones 6 weeks before study start or when they were pregnant and/or lactating. All skin samples were collected using a 4-mm-diameter skin punch. Before the biopsy, the skin was disinfected with 70% alcohol and a 4% chlorhexidine solution followed by local anaesthesia with 2% lidocaine hydrochloride. Biopsies were taken from the interscapular regions of the dogs and soaked in 2 ml saline solution (NaCl 0.9%) for at least 12 h. After the procedure, the wounds on the dogs’ skin were closed with Histoacryl® (Braun) or a Sterile Disposable Skin Stapler as per the owners’ preferences. Dogs were enrolled when *O. lupi* or *Cercopithifilaria* spp. mfs were detected in 20 µl of skin sediment and their number was recorded in individual forms.

On study day (SD) 0, dogs were weighed and their body weight recorded with the purpose of dosing the pharmaceutical product used. Twenty-two dogs meeting the inclusion criteria were included in the study and allocated to treatment group (*n* = 11; G1) or left untreated as control (*n* = 11; G2) following a random treatment allocation plan on the basis of an inclusion sequence. As a pilot investigation to test the treatment efficacy, five dogs assigned to G1 were treated four times at monthly intervals with Advocate® spot-on (Bayer Animal Health), on SDs 0, 28 (± 2), 56 (± 2), and 84 (± 2), at the minimum recommended dose rate of 2.5 mg moxidectin/kg body weight (BW) and 10 mg imidacloprid/kg BW corresponding to ≥ 0.1 ml spot-on formulation per kg BW. Dogs were treated with the appropriate pipette size according to their weight. Based on the negative results for both *O. lupi* or *Cercopithifilaria* spp. mfs obtained in animals treated in the pilot study, from SD28 onwards, the remaining six dogs in G1 were treated and assessed only once, at SD0 and SD28, respectively. The 11 animals in the control group (G2) were left untreated, but samples for diagnosis were taken at the same time point of the six animals left in G1.

### Molecular identification

Microfilariae were isolated and genomic DNA extracted using a commercial kit (DNeasy Blood & Tissue Kit, Qiagen, Germany) in accordance with the manufacturer’s instructions. Samples were molecularly processed for specific amplification and sequencing of the partial cytochrome oxidase subunit 1 (*cox*1) gene (~ 689 bp), following procedures described elsewhere using generic primers for filarioids [21]. Amplicons obtained from the skin sediments were purified using Ultrafree-DA columns (Amicon, Millipore, USA) and sequenced directly with the Taq DyeDeoxyTerminator Cycle Sequencing Kit (v.2, Applied Biosystems, USA) in an automated sequencer (ABI-PRISM 377, Applied Biosystems).

Sequences were aligned using the Geneious R9 software package (http://www.geneious.com) and compared (BLASTn) with those available in the GeneBank database (http:/ blast.ncbi.nlm.nih.gov/Blast.cgi).

### Calculation of the efficacy and statistical analyses

The percentage (%) of reduction [t] of mfs was calculated as follows: [t] = [(Ct0–Ct)/Ct0] × 100, where Ct0 is the baseline count before treatment and Ct was the count at any time point (t). Moreover, efficacy (%) = [(Ct–T)/Ct] × 100, where Ct is the mean count of mfs of the control group at X time and T is the mean count of mfs of the treated animal groups at X time, was calculated and demonstrated by Fisher’s exact test on contingency tables. Fisher’s exact test was also performed to compare and assess the risk of mf infection according to the age and sex of the animals. Statistical analysis was planned and conducted in compliance with current guidelines [41]. Statistical calculations and randomization were performed with SPSS® statistical package for Windows, version 13.0, and nQuery + nTerim 3.0 (StatSols), Statistical Solutions® Ltd. 2014, Microsoft.

## Results

**Fig. 2:**
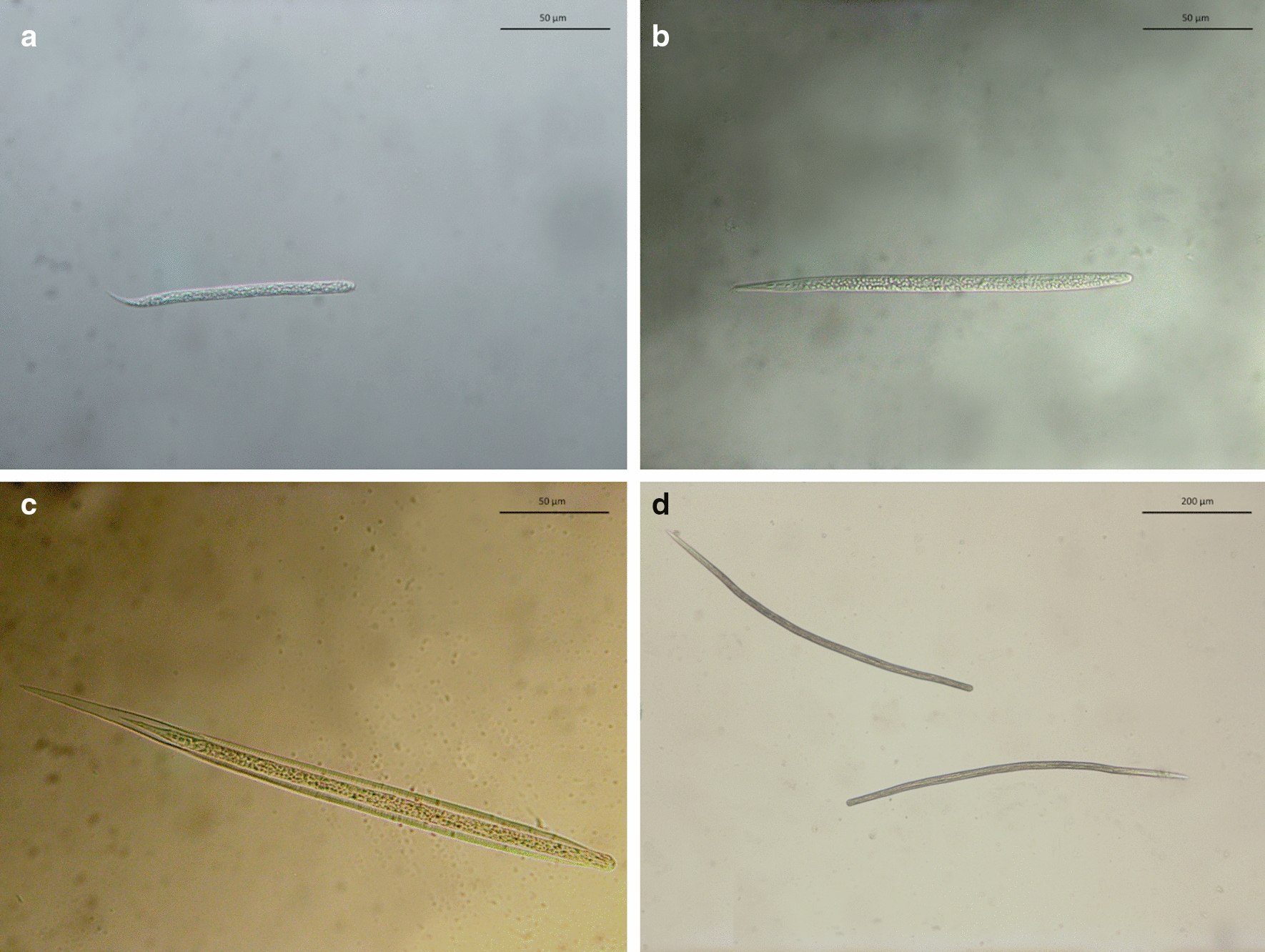
Light microscopy of microfilariae of **a** *Onchocerca lupi*, **b** *Cercopithifilaria bainae* **c** *Cercopithifilaria* sp. II sensu Otranto et al., 2013, and **d** *Cercopithifilaria grassii*

Of the 393 animals sampled, 78 (19.8%; CI 16.1‒24.2) scored positive for skin-dwelling mfs. Specifically, 72 only for *Cercopithifilaria* spp. mfs (18.3%; CI 14.7‒22.5), 2 only for *O. lupi* (0.5%; CI 0.1‒1.9) mfs, and 4 (1.0%; CI 0.4‒2.6) for both, with an overall prevalence of 19.3% (CI 15.6‒23.5) for *Cercopithifilaria* spp. and of 1.5% (CI 0.7‒3.3) for *O. lupi* (Fig. 2). Risk estimates for the infection demonstrated that males are more prone to be infected by *Cercopithifilaria* spp. mfs than females (*p* = 0.01; odds ratio: 2.1; 95% CI 1.2‒3.8). In contrast, the age of the animals evaluated was not statistically significant for mfs infection (*p* > 0.05).

At the cross-sectional study, of the 76 animals infected by *Cercopithifilaria* spp. (Fig. 2), *C. bainae* was the most prevalent species (*n* = 72; 18.3%; 95% CI 14.7‒22.5), followed by *Cercopithifilaria* sp. II (*n* = 9; 2.3%; 95% CI 1.2‒4.3) and *C. grassii* (*n* = 5; 1.3%; 95% CI 0.5‒3.0), with 9 animals being infected by one or more species of *Cercopithifilaria*. The mean number of *O. lupi* mfs (23; SD 34.76) was higher than that of *C. bainae* (mean 5.3; SD 8.4), *C. grassii* (mean 3.6; SD 3.4), and *Cercopithifilaria* sp. II (mean 3.7; SD 3.5).

At SD0, the mean number of *Cercopithifilaria* spp. mfs was 12.6 (SD: 13.9; 95% CI 3.3‒22.0) for G1 and 8.7 (SD: 6.6; 95% CI 4.3‒13.2) for G2, with no statistically significant difference among both groups (*p* > 0.05). A mean number of 19.6 (SD: 20.2; 95% CI: 5.5‒44.7) for *O. lupi* was recorded among five infected dogs in G1. At SD28 no mfs were detected in G1, therefore indicating 100% efficacy for the employed treatment after the first application. The count number of *O. lupi* and *Cerchopithifilaria* spp. microfilarie for G1 and G2 at SD0 and SD28 is reported in Table 1. Dogs did not present clinical signs suggestive for infection by *O. lupi* and/or *Cercopithifilaria* spp. The morphological identification of mfs was molecularly confirmed, and nucleotide sequences obtained from microfilarial DNA displayed 100% identity with those of *O. lupi* from Portugal (GenBank; accession number: EF521410), *C. bainae* (GenBank; accession number: JF461457), *Cercopithifilaria* sp. II (GenBank; accession number: JQ837809), and *C. grassii* (GenBank; accession number: JQ837810).

**Table 1:** Sex, age, breed, and microfilariae counting number for dogs of the treatment (G1) and control (G2) groups enrolled in this study

Study group	Serial number of dogs	Sex	Age (years)	Breed	SD0 (*no*. of larvae)	SD28 (*n*o. of larvae)
G1 (Treatment)	5*	M	> 1 to < 5	Portuguese Warren Hound	52 (*Ol*) + 29 (*C*s)	Neg
	11*	M	≥ 5	Mongrel	26 (*Ol*) + 4 (*C*s)	Neg
	13*	M	≥ 5	Portuguese Warren Hound	5 (*Ol*) + 1 (*C*s)	Neg
	18*	M	≥ 5	Portuguese Warren Hound	12 (*Ol*)	Neg
	21*	M	≥ 5	Barrocal Algarvio	3 (*Ol*) + 12 (*C*s)	Neg
	315	F	≥ 5	Pitbull	2 (*Cb*)	Neg
	346	M	≥ 5	Portuguese Warren Hound	34 (*Cb*)	Neg
	373	F	≤ 1	Anglo-French hound	3 (*Cb*)	Neg
	414	M	> 1 to < 5	Mongrel	17 (*Cb*)	Neg
	442	M	≥ 5	Grand Bleu de Gascogne	35 (*Cb*)	Neg
	450	M	≤ 1	Barrocal Algarvio	2 (*Cb*)	Neg
G2 (Control)	313	M	≥ 5	Portuguese Warren Hound	9 (*Cg*)	10 (*Cg*)
	320	M	> 1 to < 5	Portuguese Warren Hound	14 (*Cb*)	5 (*Cb*)
	323	M	> 1 to < 5	Dogo Argentino x Pitbull	24 (*Cb*)	47 (*Cb*)
	327	M	> 1 to < 5	Portuguese Warren Hound	4 (*Cb*)	Neg
	333	M	> 1 to < 5	Portuguese Warren Hound	5 (*Cb*)	3 (*Cb*)
	381	M	> 1 to < 5	Paderne	2 (*Cb*)	26 (*Cb*)
	382	M	≤ 1	Dogo Argentino	6 (*Cb*)	12 (*Cb*)
	385	M	> 1 to < 5	Dogo Argentino	12 (*Cb*)	20 (*Cb*)
	418	M	> 1 to < 5	Mongrel	5 (*Cb*)	4 (*Cb*)
	425	M	> 1 to < 5	Mongrel	13 (*Cb*)	12 (*Cb*)
	451	M	≤ 1	Paderne × Barrocal Algarvio	2 (*Cb*)	82 (*Cb*)

Microfilariae counting was performed at SD0 and reassessment at SD28 for all animals in both groups

*Ol* *Onchocerca lupi*, *Cs* *Cercopthifilaria* spp., *Cb* *Cercopthifilaria bainae*, *Cg* *Cercopthifilaria grassi*

^*^Animals included in the pilot study as they received the treatment and were assessed for mfs on SD0, SD28, SD56, and SD84, remaining negative

## Discussion

A single administration of moxidectin/imidacloprid spot-on formulation (Advocate® spot-on) was shown to be efficacious for the treatment of skin-dwelling mfs in naturally infected dogs. This has been assessed through counting mfs in the sediment of skin biopsy punches obtained pre- and post-treatment by comparison with untreated control animals positive for *Cercopithifilaria* spp. mfs. Based on the preliminary results in 5 animals, the remaining 17 animals were checked for mfs only on SD28, also considering the reluctancy of animal owners to authorize collection of multiple skin biopsies, which is a rather invasive procedure.

The efficacy of a single administration of a moxidectin/imidacloprid spot-on formulation is expected because the product is currently licensed in Europe for the hemoprophylaxis of *D. immitis* and *D. repens*. Furthermore, the product is labelled for microfilaria treatment based on the microfilaricidal efficacy of moxidectin against blood-circulating mfs. This has been demonstrated herein also against skin-dwelling mfs of *Cercopithifilaria* spp. and it is suggestive also for *O.* *lupi*. Nonetheless, since reinfection may be an issue, in endemic areas, the treatment should be kept monthly to avoid new infection.

Overall, to our knowledge this is the first evidence-based protocol for cleaning *O. lupi* mfs infections since efficacy of drugs has been mainly assessed for curing nodules [42].

Indeed, avermectins have been used for treating *O. lupi* nodules in dogs in different formulations and timing. For example, other treatment protocols previously adopted included moxidectin (i.e. 0.22 mg/kg subcutaneous injection administered, followed by two additional injections at monthly intervals), doxycycline (5 mg/kg orally twice daily for 3 months), and following monthly recheck examinations and a final moxidectin injection, and prophylactic oral heartworm therapy (400 µg/kg orally every day for 30 days) [16]. Also, ivermectin administered at 150 μg/kg/dose every 3 months was successfully used for curing nodules [43]. While previous studies conducted in Greece demonstrated that ivermectin cured and prevented infection in 23 dogs with subconjunctival onchocerciasis [44], 16 ivermectin-treated dogs in the USA showed a 67% infection recurrence [38]. This could be due to the strain of *O. lupi* circulating in the USA or to specific epidemiological conditions that are yet unknown [45].

Similarly, the moxidectin/imidacloprid spot-on formulation herein tested was shown to be efficacious against mfs of *Cercopithifilaria* spp. This study confirms a previous report which suggested the efficacy of the imidacloprid/moxidectin spot-on formulation in association with prednisone in treating *C. bainae*-caused dermatitis in the USA [26].

The prevalence of infection by *O. lupi* at the initial screening (i.e. 1.5%) was lower than that recorded in a previous study in the same geographical area (i.e. 8% [40]; 17.1% [39]). However, due to the knowledge gaps in the life cycle of this filarioid (e.g. the identity of the vector is unknown), the epidemiology of the infection remains rather obscure. On the other hand, for *Cercopithifilaria* spp. the overall prevalence herein detected (19.8%) is similar to that previously reported in the same area in Portugal (i.e. 23.5%; [23]) and in central Spain (i.e. 21.6%; [21]). Particularly, *C. bainae* was the most prevalent species, followed by *Cercopithifilaria* sp. II and *C. grassii* as reported in the same geographical area [23].

## Conclusions

Results obtained in this study indicate that moxidectin/imidacloprid spot-on formulation seems to be efficacious for the treatment of *O. lupi* and *Cercopithifilaria* spp. skin-dwelling mfs in infected dogs in a single administration. Therefore, this treatment may prove useful for the prevention of the infection caused by these little-known filarioids. Finally, assessing the microfilaricidal efficacy in dogs could also be useful in reducing the risk of *O. lupi* infection for humans.

## Data Availability

Data supporting the conclusions of this article are included within the article and its additional files. The raw datasets used and analysed during the current study are available from the corresponding author upon reasonable request.

## Abbreviations

BW: Body weight
CI: Confidence interval
GSP: Good Scientific Practice
Mfs: Microfilariae
SD: Study day

## References

[1] Hawking F, Worms M (1961). Transmission of filarioid nematodes. Annu Rev Entomol.

[2] Pennington NE, Phelps CA (1969). Canine filariasis on Okinawa, Ryukyu Islands. J Med Entomol.

[3] Schacher JF (1973). Laboratory models in filariasis: a review of filarial life-cycle patterns. Southeast Asian J Trop Med Public Health.

[4] Rani PA, Coleman GT, Irwin PJ, Traub RJ (2011). Hippobosca longipennis–a potential intermediate host of a species of Acanthocheilonema in dogs in northern India. Parasit Vectors.

[5] Pampiglione S, Rivasi F (2000). Human dirofilariasis due to *Dirofilaria* (*Nochtiella*) *repens*: an update of world literature from 1995 to 2000. Parassitologia.

[6] Capelli G, Genchi C, Baneth G, Bourdeau P, Brianti E, Cardoso L (2018). Recent advances on *Dirofilaria repens* in dogs and humans in Europe. Parasit Vectors.

[7] Dantas-Torres F, Otranto D (2020). Overview on *Dirofilaria immitis* in the Americas, with notes on other filarial worms infecting dogs. Vet Parasitol.

[8] Lefoulon E, Giannelli A, Makepeace BL, Mutafchiev Y, Townson S, Uni S (2017). Whence river blindness? The domestication of mammals and host-parasite co-evolution in the nematode genus *Onchocerca*. Int J Parasitol.

[9] Otranto D, Sakru N, Testini G, Gürlü VP, Yakar K, Lia RP (2011). Case report: First evidence of human zoonotic infection by *Onchocerca lupi* (Spirurida, Onchocercidae). Am J Trop Med Hyg.

[10] Otranto D, Eberhard ML (2011). Zoonotic helminths affecting the human eye. Parasit Vectors.

[11] Otranto D, Giannelli A, Latrofa MS, Dantas-Torres F, Trumble NS, Chavkin M (2015). Canine infections with *Onchocerca lupi* Nematodes, United States, 2011–2014. Emerg Infect Dis.

[12] Berry R, Stepenaske S, Dehority W, Almeida M, Nmathison B, Bishop H, et al. Zoonotic *Onchocerca lupi* presenting as a subcutaneous nodule in a 10-year-old girl: report of the second case in the United states and a review of the literature. Abstract Book of ASDP annual meeting, Nov 6–9, 2014, Chicago, USA.

[13] Dudley RWR, Smith C, Dishop M, Mirsky D, Handler MH, Rao S (2015). A cervical spine mass caused by *Onchocerca lupi*. Lancet.

[14] Otranto D, Dantas-Torres F, Cebeci Z, Yeniad B, Buyukbabani N, Boral OB (2012). Human ocular filariasis: further evidence on the zoonotic role of *Onchocerca lupi*. Parasit Vectors.

[15] Grácio AJ, Richter J, Komnenou AT, Grácio MA (2015). Onchocerciasis caused by *Onchocerca lupi*: an emerging zoonotic infection. Systematic Rev Parasitol Res.

[16] Otranto D, Giannelli A, Scotty Trumble N, Chavkin M, Kennard G, Latrofa MS (2015). Clinical case presentation and a review of the literature of canine onchocercosis by *Onchocerca lupi* in the United States. Parasit Vectors.

[17] Franchini D, Giannelli A, Di Paola G, Cortes H, Cardoso L, Lia RP (2014). Image diagnosis of zoonotic onchocercosis by *Onchocerca lupi*. Vet Parasitol.

[18] Colella V, Lia RP, Di Paola G, Cortes H, Cardoso L, Otranto D (2018). International dog travelling and risk for zoonotic *Onchocerca lupi*. Transbound Emerg Dis.

[19] Otranto D, Dantas-Torres F, Papadopoulos E, Petrić D, Ćupina AI, Bain O (2012). Tracking the vector of *Onchocerca lupi* in a rural area of Greece. Emerg Infect Dis.

[20] Hassan HK, Bolcen S, Kubofcik J, Nutman TB, Eberhard ML, Middleton K (2015). Isolation of *Onchocerca lupi* in dogs and black flies, California, USA. Emerg Infect Dis.

[21] Otranto D, Brianti E, Dantas-Torres F, Miró G, Latrofa MS, Mutafchiev Y (2013). Species diversity of dermal microfilariae of the genus *Cercopithifilaria* infesting dogs in the Mediterranean region. Parasitology.

[22] Otranto D, Dantas-Torres F, Giannelli A, Abramo F, Ignjatović Ćupina A, Petrić D (2013). Cutaneous distribution and circadian rhythm of *Onchocerca lupi* microfilariae in dogs. PLoS Negl Trop Dis.

[23] Cortes HC, Cardoso L, Giannelli A, Latrofa MS, Dantas-Torres F, Otranto D (2014). Diversity of *Cercopithifilaria* species in dogs from Portugal. Parasit Vectors.

[24] Ionică AM, D'Amico G, Mitková B, Kalmár Z, Annoscia G, Otranto D (2014). First report of Cercopithifilaria spp. in dogs from Eastern Europe with an overview of their geographic distribution in Europe. Parasitol Res..

[25] Latrofa MS, Dantas-Torres F, Giannelli A, Otranto D (2014). Molecular detection of tick-borne pathogens in *Rhipicephalus sanguineus* group ticks. Ticks Tick Borne Dis.

[26] Boyd M, Santoro D, Craft WF, Ginn PE, Childress AL, Wellehan JFX (2019). Dermatitis caused by autochthonous *Cercopithifilaria bainae* from a dog in Florida, USA: clinical, histological and parasitological diagnosis and treatment. Vet Dermatol.

[27] Brianti E, Otranto D, Dantas-Torres F, Weigl S, Latrofa MS, Gaglio G (2012). *Rhipicephalus sanguineus* (Ixodida, Ixodidae) as intermediate host of a canine neglected filarial species with dermal microfilariae. Vet Parasitol.

[28] Ramos RA, Giannelli A, Brianti E, Annoscia G, Cantacessi C, Dantas-Torres F (2013). Tick vectors of *Cercopithifilaria bainae* in dogs: *Rhipicephalus sanguineus* sensu lato versus *Ixodes ricinus*. Parasitol Res.

[29] Ramos RA, Giannelli A, Carbone D, Baneth G, Dantas-Torres F, Otranto D (2014). Occurrence of *Hepatozoon canis* and *Cercopithifilaria bainae* in an off-host population of *Rhipicephalus sanguineus sensu* lato ticks. Ticks Tick Borne Dis.

[30] Gabrielli S, Giannelli A, Brianti E, Dantas-Torres F, Bufalini M, Fraulo M (2014). Chronic polyarthritis associated to *Cercopithifilaria bainae* infection in a dog. Vet Parasitol.

[31] Egyed Z, Sréter T, Széll Z, Beszteri B, Oravecz O, Márialigeti K (2001). Morphologic and genetic characterization of *Onchocerca lupi* infecting dogs. Vet Parasitol.

[32] Sréter-Lancz Z, Széll Z, Sréter T (2007). Molecular genetic comparison of Onchocerca sp. infecting dogs in Europe with other spirurid nematodes including Onchocerca lienalis. Vet Parasitol..

[33] Latrofa MS, Annoscia G, Colella V, Cavalera MA, Maia C, Martin C (2018). A real-time PCR tool for the surveillance of zoonotic *Onchocerca lupi* in dogs, cats and potential vectors. PLoS Negl Trop Dis.

[34] Laidoudi Y, Bedjaoui S, Medkour H, Latrofa MS, Mekroud A, Bitam I (2020). Molecular approach for the diagnosis of blood and skin canine filarioids. Microorganisms.

[35] Giannelli A, Cantacessi C, Graves P, Becker L, Campbell BE, Dantas-Torres F (2014). A preliminary investigation of serological tools for the detection of *Onchocerca lupi* infection in dogs. Parasitol Res.

[36] Campbell B, Cortes H, Annoscia G, Giannelli A, Parisi A, Latrofa MS (2016). Paramyosin of canine *Onchocerca lupi*: usefulness for the diagnosis of a neglected zoonotic disease. Parasit Vectors.

[37] Latrofa MS, Palmisano G, Annoscia G, Pierri CR, Chandrashekar R, Otranto D (2021). Major antigen and paramyosin proteins as candidate biomarkers for serodiagnosis of canine infection by zoonotic *Onchocerca lupi*. PLOS Negl Trop Dis.

[38] McLean NJ, Newkirk K, Adema CM (2017). Canine ocular onchocerciasis: a retrospective review of the diagnosis, treatment, and outcome of 16 cases in New Mexico (2011–2015). Vet Ophthalmol.

[39] Colella V, Maia C, Pereira A, Gonçalves N, Caruso M, Martin C (2018). Evaluation of oxfendazole in the treatment of zoonotic *Onchocerca lupi* infection in dogs. PLoS Negl Trop Dis.

[40] Otranto D, Dantas-Torres F, Giannelli A, Latrofa MS, Papadopoulos E, Cardoso L (2013). Zoonotic *Onchocerca lupi* infection in dogs, Greece and Portugal, 2011–2012. Emerg Infect Dis.

[41] EMA/CVMP/EWP/81976/2010. Guideline on statistical principles for clinical trials for veterinary medicinal products (pharmaceuticals). http://www.ema.europa.eu/docs/en_GB/document_library/Scientific_guideline/2012/01/WC500120834.pdf.

[42] Cantey PT, Eberhard M, Weeks J, Swoboda S, Ostovar GA (2016). Letter to the Editor: *Onchocerca lupi* infection. J Neurosurg Pediatr.

[43] Komnenou AT, Thomas AL, Papadopoulos E, Koutinas AF (2016). Intraocular localization of *Onchocerca lupi* adult worm in a dog with anterior uveitis: A case report. Vet Ophthalmol.

[44] Komnenou A, Eberhard ML, Kaldrymidou E, Tsalie E, Dessiris A (2002). Subconjunctival filariasis due to Onchocerca sp. in dogs: report of 23 cases in Greece. Vet Ophthalmol.

[45] Rojas A, Morales-Calvo F, Salant H, Otranto D, Baneth G. Zoonotic ocular onchocercosis by *Onchocerca lupi*. Yale J Biol Med. 2021 (**in press**). PMC8223538 34211352

